# A183 SUCCINATE IN THE RELATIONSHIP BETWEEN INFLAMMATORY BOWEL DISEASE AND OBESITY IN SUCCINATE RECEPTOR DEFICIENT MICE

**DOI:** 10.1093/jcag/gwab049.182

**Published:** 2022-02-21

**Authors:** A M Arsenault, J E Nettleton, M O Otley, C Sinal, J M Connors, A Stadnyk

**Affiliations:** Dalhousie University, Halifax, NS, Canada

## Abstract

**Background:**

The incidence of Inflammatory Bowel Disease (IBD) among the obese pediatric populations is increasing. Succinate has been identified as a possible metabolite linking the two diseases. Succinate receptor 1 (SUCNR1) gene knockout (KO) mice are less susceptible to dextran sulfate sodium (DSS)-induced colitis.

**Aims:**

To determine whether succinate plays a role in mice becoming obese and colitis in obesity, and whether an obese-inducing diet would change the gut microbiota. We hypothesized that SUCNR1-KO mice would not become obese and would experience less colonic inflammation despite the diet.

**Methods:**

C57BL/6 (WT) were bred with SUCNR1-KO mice (generously provided by Amgen), and the heterozygous (HZ) F1 offspring bred to obtain F2. Two of 3 F2 litters included SUCNR1-KO mice, which were caged by sex but not genotype. These F2 mice began a high-fat/high-sugar (obese, Dytes) diet at 5 wks of age for 5 wks. Weights were recorded and stool collected. All mice then had 3% DSS replace their water, for 5 days. Mice were observed for diarrhea and occult blood. After the DSS, facility water was returned for 1 day prior to postmortem analyses. Mice were scanned using dual-energy X-ray absorptiometry (DEXA) for measures of fat, lean and fat mass, and bone density. Their colons were resected and fixed for histopathology. Stool was banked frozen until processed for sequencing using 16S Ribosomal primers.

**Results:**

Three F2 litters were comprised of ratios of HZ:WT:SUCNR1-KO of 14:7:2. SUCNR1-KO mice (n=2 female) had a greater increase in weight compared to other genotypes during the obesity-induction phase. Weight loss during the DSS phase was similar across all genotypes. All mice had blood in their stool. DEXA measures did not differ between genotypes. All genotypes of mice had inflamed colons.

**Conclusions:**

SUCNR1-KO mice are not resistant to obesity, nor from colitis when consuming the obese diet, outcomes that do not support our hypothesis. It remains to be determined whether the diet alters the microbiome resulting in SUCNR1-KO mice being suscueptible to colitis. Microbial sequencing is underway.

**Funding Agencies:**

IWK Health Project Grant

